# Effects of Temephos (Abate^®^), Spinosad (Natular^®^), and Diflubenzuron on the Survival of Cyclopoid Copepods

**DOI:** 10.4269/ajtmh.21-0818

**Published:** 2022-01-24

**Authors:** Ryan Grunert, Erin Box, Kayla Garrett, Michael Yabsley, Christopher Cleveland

**Affiliations:** ^1^ Southeastern Cooperative Wildlife Disease Study, Department of Population Health, College of Veterinary Medicine, University of Georgia, Athens, Georgia;; ^2^ Warnell School of Forestry and Natural Resources, University of Georgia, Athens, Georgia

## Abstract

*Dracunculus medinensis*, also known as the African Guinea worm, is the causative agent of dracunculiasis and the focus of the global Guinea Worm Eradication Program (GWEP). Transmission of *D. medinensis* to humans occurs primarily by drinking water containing cyclopoid copepods infected with third-stage *D. medinensis* larvae. A common intervention to interrupt transmission and decrease the number of copepods in infected water bodies is the application of the organophosphate larvicide Abate^®^ (temephos). However, the use of alternative compounds to help decrease copepod populations would be beneficial to the GWEP. We compared the immobilization of copepods by three compounds: Abate, Natular^®^ (spinosad), and diflubenzuron. Our results confirm that neither diflubenzuron nor Natular immobilized copepods as quickly or as effectively as Abate. However, doubling or tripling the suggested concentration of Natular resulted in immobilization rates similar to Abate over 72 hours of continuous exposure. Further research on the possible effects of higher concentrations of Natular on the environment and nontarget organisms is necessary to determine whether this compound can be used safely to control the copepod population.

## INTRODUCTION

Dracunculiasis (Guinea worm disease) is a neglected tropical disease that historically caused significant morbidity in people living in previously endemic communities. This disease is caused by infection with the parasitic nematode, *Dracunculus medinensis*,^1^^–^^4^ which is transmitted to definitive hosts when cyclopoid copepod intermediate hosts infected with third-stage guinea worm larvae (L3s) are ingested from contaminated drinking water.^2^^,^^4^ The global Guinea Worm Eradication Program (GWEP), started by the CDC in 1980 and led by The Carter Center since 1986, has been extremely successful in reducing the number of human cases by > 99.99% (> 3.5 million in 1980 to 27 cases in 2020 [provisional as of January 2021]).^5^^,^^6^ The lack of a preventative vaccine or medicinal treatment of dracunculiasis highlights the importance of interventions targeting community-focused education and tactics that can prevent ingestion of copepods and break the transmission cycle of *D. medinensis*.^6^ Common intervention methods targeting copepods that the GWEP has used include provisioning of filters and drinking straws equipped with filters to screen copepods out of collected drinking water and the application of chemical larvicides to infested water bodies.^1^^,^^7^

Currently, most water sources in guinea worm endemic countries are treated with the organophosphate larvicide Abate^®^ (temephos). Abate was introduced as a mosquito larvicide, but is also effective for controlling *D. medinensis* transmission because of its high toxicity to copepods and low toxicity to mammals when used in small doses at application sites.^7^^,^^8^ Abate inhibits cholinesterase production and after 12 hours of exposure at the field approved application rate of 1 mg/L, cholinesterase levels in copepods begin to reduce and they lose their ability to feed and swim normally.^7^ Within 72 hours, the depleted cholinesterase levels cause paralysis and the copepods settle to the bottom of the water system and ultimately perish.^7^

The Abate formulation currently used by the GWEP is a liquid 50% emulsifiable concentrate. To accurately apply Abate at the appropriate concentration, the volume of water in the targeted water source must be calculated. This can be difficult in some African water bodies due to unfavorable weather conditions (rainy or dry season) or low accessibility, and is impossible in flowing bodies of water.^7^^,^^9^

Natular^®^ (spinosad) is a mosquito larvicide that is currently used to control common mosquito vector species in the genera *Aedes*, *Anopheles*, and *Culex*.^10^^–^^13^ The active component of Natular, spinosad, is a bacterially derived mixture of neurotoxins spinosyn A and D produced by aerobic fermentation of *Saccharopolyspora spinosa*.^10^^,^^13^ Natular acts on the postsynaptic nicotinic acetylcholine and gamma-aminobutyric acid (GABA) receptors, causing rapid excitation of the nervous system, paralysis, and death of the invertebrate.^12^ This mechanism of action makes Natular relatively slow acting, with maximum cumulative mortality taking up to 72 hours to occur.^12^ Natular has a favorable environmental profile with low mammalian toxicity and is commercially available in eight formulations.^10^ When used in recommended doses, spinosad-based products can affect a large range of nontarget invertebrates while being innocuous for fish and other aquatic vertebrates.^13^^,^^14^

Diflubenzuron is an insect growth regulator (IGR) that is commonly used on forest and field crops to control insects, including the forest tent caterpillar (*Malacosoma disstria*), gypsy moth (*Lymantria dispar*), and dipteran larvae.^15^^–^^17^ Diflubenzuron inhibits chitin synthase 1 (CHS1), which prevents catalyzation of the production of chitin in the affected organism.^17^ This mechanism of action renders exposed insects and crustaceans unable to shed their exuvium during ecdysis, resulting in mortality.^15^^,^^16^^,^^18^ The chitin-inhibiting effect of diflubenzuron has been shown to be effective on impeding the development of calanoid and harpacticoid copepods.^15^^,^^16^^,^^18^^,^^19^ Similar to Natular, this mechanism of action can affect a large range of nontarget invertebrates, but also has little effect on other aquatic vertebrates.^16^^,^^17^

## MATERIALS AND METHODS

Laboratory-raised copepods (from wild-caught stock in June and July of 2019 from Athens, Georgia) of the *Macrocyclops* genus were used to test all three compounds. Copepod identification was confirmed by sequence analysis of the partial cytochrome oxidase subunit 1 (COI) gene (GenBank accession numbers: MW522586 and MW522587).^20^ The adult copepods were maintained in laboratory colonies containing dechlorinated water and hay infusion.^8^^,^^21^ First instar mosquito (*Anopheles stephensi*) larvae were included in this study as a positive control. All trials were replicated five times and kept at an average room temperature of 21°C. Dissolved oxygen (DO), water temperature, and pH were recorded for all trials.

### Sensitivity test.

**Figure 1.**
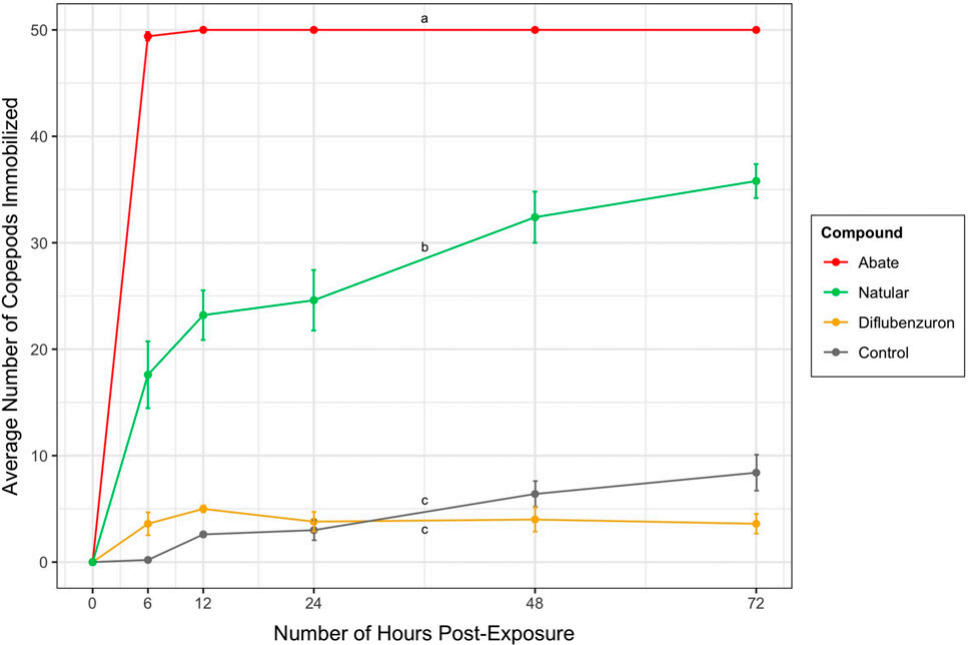
Average number of copepods immobilized (from 50 copepods/1 L water) by chemical treatment (Abate, Natular, Diflubenzuron, Control) over 72 hours. The concentrations used were 1 ppm for Abate, 7.14 ppm for Natular, and 10 ppm for Diflubenzuron. Lines represent average number of copepods immobilized, and error bars represent standard error. Significant differences between chemical treatments determined by Tukey post-hoc contrasts are indicated by “a,” “b,” and “c.” This figure appears in color at www.ajtmh.org.

The formulations of each compound tested in these experiments were Abate 50% emulsifiable concentrate (BASF Corporation, Ludwigshafen, Germany), Natular DT Bi-layer tablets (Clarke^®^ Mosquito Control Products, St. Charles, IL), and a single component mixture of diflubenzuron in methanol. This Abate formulation was chosen due to its current use in the GWEP, and the other formulations were chosen based on their ease of accessibility and current availability. The first experiment was a sensitivity test directly comparing the effects of all three compounds on copepods. Each test was performed in beakers filled with 1 L of dechlorinated water and 50 adult cyclopoid copepods. The tests were started (0 hours) when the per label application concentration of each compound was added to the beaker (Figure 1). The concentrations were 1 ppm for Abate, 7.14 ppm for Natular, and 10 ppm for diflubenzuron. These concentrations were prepared in the beakers by adding the proportionate amount of each compound required to create the suggested concentrations in 1 L of water. Copepods were considered immobilized if they were observed to have no visible movement and did not react to slight agitation with a pipette. Trials were performed for 72 hours and immobilized copepods were counted by filtering at 6, 12, 24, 48, and 72 hours after exposure. The copepods were filtered by pouring the water through a 100 um filter, counting, and reconstituting them in the same water after counting immobilization. The previously stated concentrations of Abate and Natular were each tested on 250 *A. stephensi* first instar larvae in petri dishes filled with 50 mL of dechlorinated water.

### Additional testing of Natular concentrations.

Preliminary results after the first experiment indicated that Abate was effective at immobilizing copepods and diflubenzuron was not. Natular did not immobilize as many copepods as Abate, but it was determined to have immobilization potential if the concentration was increased. Therefore, a second experiment was conducted to examine the efficacy of double and triple the suggested concentrations (14.28 ppm and 21.42 ppm) of Natular on copepods. Testing an increased concentration of Abate was determined not necessary because the efficacy of the compound on copepods could not have been improved based on the results from our already completed sensitivity test.

### Statistical analyses.

Statistical analyses were performed in R.^22^ We fit a one-way repeated measures analysis of variance model using the function *car*^23^ to determine whether the number of hours post-exposure (6, 12, 24, 48, 72 hours) to each chemical treatment (Abate, Natular, and diflubenzuron) had a statistically significant effect on the number of copepods immobilized. A one-way repeated measures analysis of variance model was similarly done on the additional testing of Natular to determine whether the number of hours post-exposure had a statistically significant effect on the number of copepods immobilized with each concentration of Natular used. We used Tukey post-hoc contrasts to determine significant differences between chemical treatments for the first experiment and Natular concentrations for the second experiment.

## RESULTS

The sensitivity test (*Sensitivity test section*) showed that Abate immobilized more copepods than Natular and diflubenzuron (Figure 1) when used at labeled/appropriate concentrations. Application of Abate resulted in immobilization of 98% of adult copepods within 6 hours, and the copepods remained immobilized throughout the duration of the test. Natular immobilized an average of 72% of copepods after the full 72 hours. Diflubenzuron only immobilized an average of 8% of copepods.

The number of hours post-exposure had a statistically significant effect on the number of copepods immobilized using each chemical treatment, F (5, 15) = 3.5893, *P* < 0.05 (Table 1). Compared with the control, Natular does have significant effect on copepod immobilization, but it was not as effective as Abate (Figure 1). Abate and Natular immobilized significantly different numbers of copepods (Figure 1, *P* < 0.05). Abate and Natular both successfully immobilized all positive control *A. stephensi* larvae within 24 hours (data not shown).

**Figure 2.**
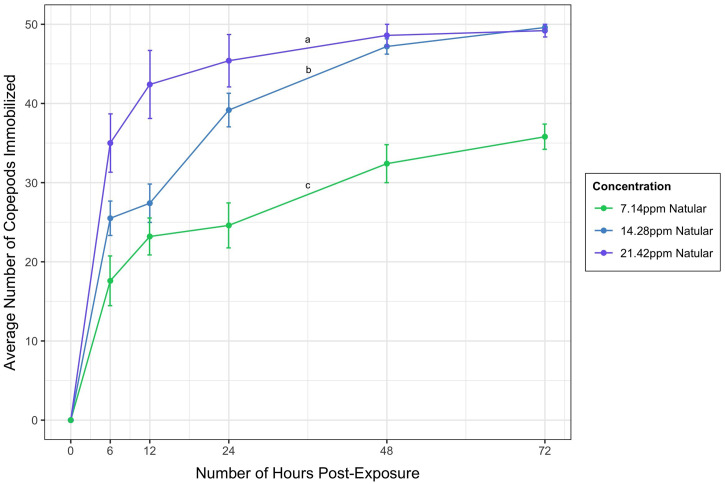
Average number of copepods immobilized (from 50 copepods/1 L) by three different concentrations of Natular (7.14 ppm, 14.28 ppm, and 21.42 ppm). Lines represent average number of copepods immobilized, and error bars represent standard error. Significant differences between concentrations of Natular determined by Tukey post-hoc contrasts are indicated by “a,” “b,” and “c.” This figure appears in color at www.ajtmh.org.

The results from the second experiment (*Additional testing of Natular concentrations section*) indicate that increased concentrations of Natular resulted in almost total immobilization of all 50 copepods after the full 72 hours (Figure 2). However, the highest concentration of Natular resulted in a faster average immobilization rate than the double concentration. The number of hours post-exposure had a statistically significant effect on the number of copepods immobilized with each concentration of Natular used, F (5, 10) = 34.805, *P* < 0.001 (Table 2). Each concentration of Natular tested immobilized a significantly different number of copepods (Figure 2, *P* < 0.05). The only exceptions (intersections of the error bars) were between 7.14 ppm and 14.28 ppm of Natular at 12 hours post-exposure, and between 14.28 ppm and 21.42 ppm at both 48 and 72 hours post-exposure.

## DISCUSSION

The objective of this study was to compare the abilities of Natular, diflubenzuron, and Abate to immobilize cyclopoid copepods. Our data indicate that within a shorter time period, Abate immobilized significantly more copepods than both Natular and diflubenzuron in a laboratory setting.

These results further support that Abate is an effective option for chemical control of *D. medinensis*-infected copepods. The suggested dose of Natular (7.14 ppm) did not immobilize copepods as quickly or as effectively as Abate (1 ppm) but did reach complete immobilization of almost all 50 copepods when applied at double (14.28 ppm) or triple (21.42 ppm) the suggested concentration. These results indicate that Natular can be used at higher concentrations to control copepods. Diflubenzuron was not effective at immobilizing copepods; however, the copepods that were tested in this study were adults. Due to diflubenzuron’s specific mechanism of action on the molting process, this compound would likely be more effective on nauplii during their growth to the adult stage. Further testing on cyclopoid nauplii would be needed to conclude if this compound has potential as a possible control intervention of copepod populations in general, which would also be beneficial for the GWEP, as it has been shown to be effective on calanoid and harpacticoid nauplii.^15^^,^^19^

One aspect of copepod control that has not been investigated is the effect of Abate or other compounds on the *D. medinensis* larvae within copepods. For classical transmission through ingestion of water, the immobilization of these infected copepods makes them unlikely to be ingested or gathered when water is collected. However, it could make it easier for fish or frog transport and paratenic hosts to consume them.^6^^,^^24^^,^^25^ This has the potential to facilitate transmission to these aquatic hosts. Further research on this possibility would be beneficial as it is also possible that *D. medinensis* larvae quickly die after their copepod intermediate host is immobilized.

Natular DT tablets were developed to treat drinking water containers for over 60 consecutive days with a slow dissolve rate. However, the use of high concentrations of Natular to control infected copepod populations could have negative effects on other organisms that either live in or consume the water. Using high concentrations in the environment could have a detrimental effect on nontarget invertebrates, as spinosad-based compounds can affect an extensive range of invertebrate species.^12^^,^^14^ This need for high concentrations also brings the cost-effectiveness of the compound into question. Natular DT tablets typically cost anywhere from $12 to $17 (U.S. dollars) for 12 tablets, thus extensive use of the tablets in multiple water bodies or drinking water containers can become expensive. Seven other formulations of Natular are available and could be tested to see whether they are useful or better suited for immobilizing copepods in either a laboratory or field setting.

The copepods used in this study were collected from Georgia which, although unlikely, may react differently to the compounds tested compared with African copepods. Also, these compounds were tested in water with lower temperatures (20–22°C) than what is normally observed in summer months in Chad, Africa (∼28°C). Of note, spinosad has been shown to have higher efficacy when applied at higher temperatures, so it is possible that effectiveness in the field may be higher than observed in this study.^26^^,^^27^ Furthermore, the beakers that were used in this study did not incorporate substrate and restricted the copepods movement with a smaller volume. Wild copepods have much larger volumes of water in which they can move, and they may also flee into the substrate of waterbodies when disturbed. This could have an effect on the efficacy of the compound applied to that water body. This, coupled with the difficulty of accurately measuring and applying a compound in the field, can increase the possibility of underdosing an infected water body. There is currently a lack of information on this topic, which warrants further studies on the chemical control of wild cyclopoid copepods for the GWEP.

In conclusion, there are still a variety of factors to investigate regarding the use of Natular and diflubenzuron to decrease copepod populations. Our study confirmed the superior performance of Abate in immobilizing copepods. The use of Abate to control copepods remains a critical intervention interrupting the transmission of Guinea-worm. Future research on any possible acquired resistance to applied chemical treatments in Chadian copepod populations would greatly benefit the GWEP.

**Table 1:** Univariate type III repeated-measures ANOVA results of copepod immobilization (from 50 copepods/1 L) using three different chemical compounds (Abate, Natular, and diflubenzuron) and including the control over 72 hours assuming sphericity

Predictor	Sum of squares	Num *df*	SSE	Den *df*	F value	*P* value
(Intercept)	7,476.5	1	6,002.4	3	3.7368	0.14872
Hours	1,609.4	5	1,345.2	15	3.5893	0.02468*

ANOVA = analysis of variance; SSE = error sum of squares.

* Indicates statistically significant difference (*P* < 0.05).

**Table 2:** Univariate type III repeated-measures ANOVA results of copepod immobilization (from 50 copepods/1 L) using three different concentrations of Natular (7.14 ppm, 14.28 ppm, and 21.42 ppm) over 72 hours assuming sphericity

Predictor	Sum of squares	Num *df*	Error SS	Den *df*	F value	*P* value
(Intercept)	16,356	1	644.94	2	50.722	0.01915
Hours	4,048	5	232.61	10	34.805	< 5.2e-06*

ANOVA = analysis of variance

* Indicates statistically significant difference (*P* < 0.001).
